# Modelling the Effect of Process Parameters on the Wet Extrusion and Spheronisation of High-Loaded Nicotinamide Pellets Using a Quality by Design Approach

**DOI:** 10.3390/pharmaceutics11040154

**Published:** 2019-04-01

**Authors:** Eva-Maria Theismann, Julia K. Keppler, Martin Owen, Karin Schwarz, Walkiria Schlindwein

**Affiliations:** 1 Division of Food Technology, Kiel University, Heinrich-Hecht-Platz 10, 24118 Kiel, Germany; etheismann@foodtech.uni-kiel.de (E.-M.T.); jkeppler@foodtech.uni-kiel.de (J.K.K.); 2 Insight by Design Ltd., Stevenage SG2 8SB, UK; martin.owen.insight@gmail.com; 3 Leicester School of Pharmacy, De Montfort University, Leicester LE1 9BH, UK

**Keywords:** wet extrusion-spheronisation, quality by design, design of experiment (DoE), niacin, controlled release

## Abstract

The aim of the present study was to develop an alternative process to spray granulation in order to prepare high loaded spherical nicotinamide (NAM) pellets by wet extrusion and spheronisation. Therefore, a quality by design approach was implemented to model the effect of the process parameters of the extrusion-spheronisation process on the roundness, roughness and useable yield of the obtained pellets. The obtained results were compared to spray granulated NAM particles regarding their characteristics and their release profile in vitro after the application of an ileocolon targeted shellac coating. The wet extrusion-spheronisation process was able to form highly loaded NAM pellets (80%) with a spherical shape and a high useable yield of about 90%. However, the water content range was rather narrow between 24.7% and 21.3%. The design of experiments (DoE), showed that the spheronisation conditions speed, time and load had a greater impact on the quality attributes of the pellets than the extrusion conditions screw design, screw speed and solid feed rate (hopper speed). The best results were obtained using a low load (15 g) combined with a high rotation speed (900 m/min) and a low time (3–3.5 min). In comparison to spray granulated NAM pellets, the extruded NAM pellets resulted in a higher roughness and a higher useable yield (63% vs. 92%). Finally, the coating and dissolution test showed that the extruded and spheronised pellets are also suitable for a protective coating with an ileocolonic release profile. Due to its lower specific surface area, the required shellac concentration could be reduced while maintaining the release profile.

## 1. Introduction

Advancements in drug delivery systems were and are highly present in the pharmaceutical and food research area. Novel approaches include macro-, micro- and nanoparticulate systems as well as uniparticulate or multiparticulate systems [1]. Nanoparticulate systems are often used for enhancing the bioavailability or solubility of poorly soluble compounds e.g., by coaxial electrospraying [2] or liposomes [3]. However, for high daily doses, nanosystems could lead to high weight of the final formulation. Therefore, the improvement of micro and macro systems with high drug loads is of recent interest. Macro or micro particulate systems can also provide an enhanced bioavailability [4,5,6] and/or a side-specific delivery in the human intestine. Pelletisation techniques are playing a leading role in the field of drug delivery systems. Pellets are small, free flowing, spherical particles with a diameter from 0.5–2 mm. Pellets, as drug delivery systems, provide technological as well as therapeutic advantages in comparison to uniparticulate systems such as simplicity of coating, less bowel irritation, uniform distribution in the gastrointestinal tract and decreased peak plasma fluctuations [1,7,8]. Fluid-bed processes and wet extrusion-spheronisation provide high drug load capacity and uniformity of pellets [1,9,10] including moderate to highly water-soluble compounds such as nicotinamide (NAM). NAM is one active form of the vitamin niacin. A colon-targeted delivery of NAM can lead to beneficial effects on the host microbiome [11]. Due to the high bioavailability of NAM and its high absorption rate in the upper intestine, a protective coating is suitable for a targeted delivery in the ileocolonic region. In a previous study, the topical delivery to the colon area of NAM was achieved by application of an adapted shellac coating. Shellac is a natural resin from the lac insect *Laccifer lacca*. It is often used as pH-sensitive enteric coating due to its acid character and its high dissolution pH value of about 7.3 [12,13,14]. However, an adapted subcoating to the characteristics of the encapsulated compound can be advantageous for a targeted release [14,15]. In recent publications, shellac was used as a coating for a nanocomposite with a colon targeted release using a modified electrospraying process [16,17]. Additionally, shellac can be used for food as edible coatings [18,19,20] and/or nutraceuticals (e.g., niacin) because of its generally recognized as safe (GRAS) status and its legal approval as a food additive [21]. The NAM cores were produced by continuous spray granulation, which fulfilled the requirements for the application of a functional coating, such as sphericity and compactness. However, the useable size range was rather narrow (315–400 µm), which led to a cost- and time-intensive production process. Furthermore, a rather thick coating layer was required to achieve the desired release profile [15]. Therefore, NAM cores produced by wet extrusion-spheronisation process will be compared to the spray granulated cores in this study as an alternative process. The requirements for the NAM cores correspond to the definition of pellets, which are favored due to their small particle size (<2 mm) and their ability to pass the pylorus independently from gastric emptying, which lead to smaller individual variations. Additionally, the round shape is ideal for coating and the risk of dose dumping is lower compared to a coated single unit/tablet [7]. The aim of the present study was therefore to modulate the extrusion/spheronisation process in order to produce highly loaded NAM pellets and to compare their properties to spray granulated NAM pellets. A quality by design (QbD) approach, which is often applied in the pharmaceutical industry to guarantee the product quality [22,23] was used to define the quality target product profile (QTPP) with its critical quality attributes (CQAs). The effect of the critical material attributes (CMAs) and the critical process parameters (CPPs) on the CQAs was determined by performing design of experiments (DoEs) for the extrusion and spheronisation processes, respectively. For the wet extrusion-spheronisation process a high drug load of 80% NAM combined with 20% MCC (Avicel PH101) was used. MCC is known as the “golden standard” for extrusion and spheronisation processes. It is characterised by its high water absorption and holding capacity, which improves the wetted mass plasticity and the spheronisation [24,25]. Finally, the release profile of the coated extrudates were compared with coated spray granulates.

## 2. Materials and Methods

### 2.1. Materials

For wet extrusion and spheronisation processes, nicotinamide (NAM) were purchased from SternVitamin (Ahrensburg, Germany) and microcrystalline cellulose (MCC) Avicel Type PH 101 was ordered from FMC Health and Nutrition (Philadelphia, PA, USA). For the following coating process, shellac solution (SSB Aquagold) was obtained from Stroever Schellack Bremen (Bremen, Germany). Citric acid monohydrate was obtained from Jungbunzlauer (Basel, Switzerland) and C* Dry maltodextrin 01915 from Cargill (Minneapolis, MN, USA). Other chemicals used for dissolution tests were purchased from Carl Roth (Karlsruhe, Germany).

### 2.2. Methods

#### 2.2.1. Pellet Preparation

##### Micronisation

In order to avoid lumps, the batch of NAM was micronized using a milling machine U5-0024 from Quadro Engineering Inc. (Waterloo, ON, Canada). The square impeller and the screen 7L094R (=2.39 mm) were used.

##### Extrusion and Spheronisation

**Figure 1:**
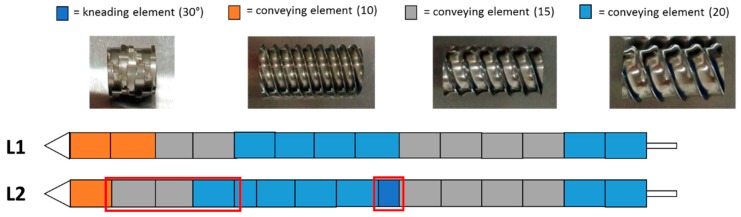
Configuration of the two different screw designs (L1 and L2) used in the present extrusion DoE. The red boxes highlight the differences between the L1 and L2 configurations.

The wet extrusion was applied using the twin-screw extruder nano 16 from Leistritz (Somerville, MA, USA) with a 1.0 mm diameter die. Prior to the extrusion, 1000 g of micronized NAM and MCC, in a ratio of 80%:20% (*w*/*w*), were dry blended in a V-shaped blender (Pharmatech, Birmingham, UK) for 20 min and filled in a single screw hopper (Brabender FlexWall, Drisburg, Germany) which fed the powder mixture into the extruder. Distilled water was used as binder liquid and was fed using a peristaltic pump (Watson-Marlow Pumps, Type 120S, Falmouth, Cornwall, UK) at 4 rpm (feed rate of 1.74 g/min). The potentially critical factors identified for the extrusion process were the screw configuration, screw speed and powder feed rate. In the present study, the hopper speed was varied to achieve an altered solid-to-liquid ratio. Therefore, a calibration curve of the powder feed rate corresponding to the hopper speed was done (powder feed rate (g/min) = 0.0382 × hopper speed − 3.6991). The used solid feed range varied between 5.3–7.9 g/min, which resulted in a liquid/solid ratio of 0.33–0.22. The screw configurations used are shown in Figure 1. After the extrusion process, the extrudates were spheronised using a spheroniser (made in house, De Montfort University, Leicester UK) with cross-hatched rotation plate with a diameter of 12 cm. The potentially critical factors identified for the spheronisation process were rotation speed, time and mass load. Two spheronisation processes were carry out for every extrusion run. The spheronised pellets were dried at 40 °C in a drying oven over night.

##### Spray Granulation of NAM

For spray granulation of NAM, a ProCell fluidized bed granulator with Vario 3 was used (Glatt Ingenieurtechnik, Weimar, Germany). For a high NAM load and a continuous process with a high useable yield, the following conditions were chosen after the conduction of preliminary experiments (not shown). First, 30% (*w*/*w*) nicotinamide including 4% (*w*/*w*) of hydroxypropyl methylcellulose (HPMC) were dissolved in tap water. The solution was sprayed through a bottom-spray nozzle with an increasing spraying rate of 5–24 g/min. The conditions of the process were the following: inlet air temperature: 80–90 °C, product temperature: 50 °C, air rate: 80–90 m^3^/h and an atomizing air pressure of 2–2.8 bar.

##### Fluidized Bed Coating

For application of a coating, a fluidized bed coater (MiniGlatt, Glatt Ingenieurtechnik GmbH, Binzen, Germany) was used. The applied coating consisted of an inner shellac coating, an intermediate citric acid coating and an outer shellac coating in accordance to previous experiments [15] to achieve an ileocolon targeted delivery of NAM. Therefore, an inner shellac layer was applied with a weight gain (w.g.) of 20%, followed by a citric acid coating, which generated a citric acid w.g. of 0.65% and a w.g. of 5.85% of maltodextrin, which was used as binder. Afterwards, an outer shellac coating (7.5% w.g.) was applied. The calculated coating w.g. were based on the coating levels for coated NAM cores produced by spray granulation (see below) including a roughness factor of 54%, which doubled the calculated coating mass application. The triple coating was sprayed onto 80 g extruded and spheronised NAM: MCC pellets under the following conditions: inlet air pressure of 0.4–0.55 bar, atomizing air pressure of 0.55–0.70 bar, inlet air temperature of 37–42 °C and a spraying rate of 0.5–0.95 g/min. In the end, the coated pellets were dried in a drying oven at 50 °C for 1 h.

##### Calculation of the Coating Level and Coating Process

The coating level (CL) [mg/cm^2^] is determined as the mass of shellac (*m_shellac_* in mg) applied to the pellet surface (*A* in cm²): $CL=\frac{m_{shellac}}{A}$ as described by [26]. *M_shellac_* is the difference between the mass of the sample (*m_sample_* in mg) and the mass of the cores (*m_cores_* in mg) in the sample. The mass of the cores in the sample was calculated to the following equation:

$$m_{cores}=100\frac{m_{NAM\;sample}}{c_{uncoated\;pellets}},$$

whereby *m_NAM sample_* is the NAM mass in the sample (mg) and *c_uncoated pellets_* the NAM content of uncoated pellets (%). The surface area of the sample was calculated from the average radius and the number of pellets (n): $A=4\pi r^{2}n$. The number of the pellets is the relation of *m_cores_* to *m_uncoated pellet_*. The average mass of pellets was calculated from 500 pellets. The average radius was measured by particle size measurements by laser diffraction using a using the dry feeder of the particle analyzer (Horiba LA-950V2, Retsch Technology GmbH, Haan, Germany) with vacuum-driven forced ejection, a vibration power alteration of 120 and a compressed air pressure of 0.3 MPa. A refractive index of 1.47 was used for NAM. The NAM content, after the application of the coating layer, was determined spectrophotometrically at 262 nm after dissolution of about 50 mg pellets in 100 mL Soerensen phosphate buffer at a pH value of 7.4 (Helios gamma, Thermo Fischer Scientific, Waltham, MA, USA).

#### 2.2.2. Extrudate Characterization

##### Process Yield

During every run, the process yield, which was defined as the extruded mass per minute (g/min), was examined by weighing. The standard deviation of three measurements was calculated to control the process variability.

##### Moisture Content

To measure the moisture content during process, freshly produced extrudates were analysed before the spheronisation process. About 2.5 g sample were weighed before and after drying at 105 °C in drying oven for 24 h. The experiments were done in triplicate. The water content is expressed as the percentage of the total weight of the wet mass.

#### 2.2.3. Pellet Characterization

##### Pellet Size Distribution—Sieve Analysis

The sieve analysis gives information about the particle size distribution after the extrusion-spheronisation process. The dried pellets of two spheronisation processes were sieved in a sieve shaker AS 200 control (Retsch GmbH, Haan, Germany) using sieve sizes of 2, 1.4, 1, 0.5 and 0.25 mm to separate the pellets according to their size. The amplitude was set to 0.5 mm and the total sieve time to 3 min. Afterwards, the per cent weight of the fractioned pellets was calculated.

The particle size analysis of spray granulated NAM pellets was done by Glatt Ingenieurtechnik GmbH (Weimar, Germany) using a Camsizer XT (Retsch Technology, Haan, Germany).

##### Pellet Shape—Photo Image and Scanning Electron Microscopy (SEM)

The shape of the spheronised pellets (n ≥ 6) was evaluated by optical inspection and by SEM. For SEM, pellets with a diameter between 1 mm and 1.4 mm were prepared on a holder with carbon Leit-tabs (Agar Scientific, Stansted, UK). Before examination in a Zeiss EVO 15 (Carl Zeiss Ltd., Cambridge, UK) at an accelerating voltage of 10 kV, microcapsules were sputter-coated with a layer of 15 nm gold using a Quorum Q150RS (Quorum Technologies Ltd., Laughton, UK) rotary pumped sputter coater. The pellets were categorical evaluated regarding their roundness and their roughness of the surface: roundness (category 1–5): 1 = irregular (like agglomerates); 3 = spherical; 5 = dumbbell-shaped or strains; roughness (category 1–5): increasing category for increasing roughness.

For SEM pictures of the spray granulated NAM pellets were prepared and analysed as described in [15].

##### Dissolution Testing after Application of Coating

The in vitro drug release study was carried out using a standard dissolution paddle apparatus at 100 rpm and 37 °C (DT 70, Pharmatest Group, Hainburg, Germany) [27]. The experiment was conducted in duplicate with 0.5 g of coated extrudated NAM: MCC pellets in 250 mL of the dissolution medium. During the experiments the amount of released NAM was recorded by UV/vis spectrophotometer (Helios γ, Thermo Fisher Scientific, Waltham, MA, USA) at the wavelength of 262 nm. The following dissolution media were used to simulate the human gastrointestinal tract from stomach to ileum to evaluate the release profile: Simulated gastric fluid (pH 1.4) was adjusted to USP gastric fluid without pepsin [28], citrate buffer (pH 4.5) was modified to [29] and phosphate buffer (pH 6.8 and 7.4) was prepared according Sorensen’s buffer. To calculate the percentage of released vitamin, the maximum NAM amount was determined by stirring (100 rpm) of 0.5 g coated NAM extrudates in 250 mL phosphate buffer at pH 7.4 for 1.5 h at 37 °C in the above-mentioned dissolution tester. The NAM concentration of the solution corresponded to 100% NAM release.

#### 2.2.4. Quality by Design

##### Quality Target Product Profile (QTPP) and Critical Quality Attributes (CQAs)

The target dosage form is a pellet for oral administration. It should have a uniform and spherical shape, a smooth surface and a narrow size distribution for an appropriate application of an additional coating in a further step. The size should be smaller than 2 mm to be able to pass the pylorus independently from gastric emptying. Furthermore, the vitamin load should be high to prevent a high number of capsules, which have to be swallowed to reach the daily dose.

The following CQAs were determined based on previous experiments: vitamin load: ≥ 80%; a roundness of category 2.0–4.0; a roughness of category ≤ 2 and a maximal useable yield (pellets with a diameter between 1 mm and 2 mm and an acceptable roundness).

##### Critical Material Attributes (CMAs) and Critical Process Parameters (CPPs)

The most critical material attributes are the high water solubility of nicotinamide (1000 g/L) and the corresponding vitamin to binder ratio. For these studies, the NAM:MCC ratio was kept constant, 80%:20% (*w*/*w*). The influence of potentially CPPs such as powder mixture feed rate, screw speed and screw configuration (for the extrusion process) and rotation speed, rotation time and mass load (for the spheronisation process) on the CQAs was investigated. The identification of CMAs and CPPs were based on preliminary risk assessment and prior knowledge. Note: CMA is not an ICH (International Conference for Harmonisation) term but it is used here to describe the quality attributes of the materials.

##### Design of Experiments (DoE)

The effects of the CPP’s were determined using a DoE approach using the statistical software JMP Pro (SAS Institute, Cary, NC, USA). For the extrusion process, two 22 full factorial designs were determined for two different screw designs, which were already shown in Figure 1. Therefore, 12 experiments were run in total, including 2 replicates of the central point for every investigated DoE. For the spheronisation process, a 2^3^ full factorial design with 10 experiments, including 2 replicates for the central point, were performed. An overview of every single experiment is listed in Table 1 and Table 2. Variations in the moisture content of the extruded mass were set by changing the hopper speed from 250 to 270, which resulted in a powder feed rate from 5.8 g/min to 6.6 g/min according to the calibration curve (powder feed rate (g/min) = 0.0382 × hopper speed − 3.6991).

## 3. Results and Discussion

### 3.1. Preliminary Tests: Establishing the Range of Solid-to-Liquid Ratio

**Figure 2:**
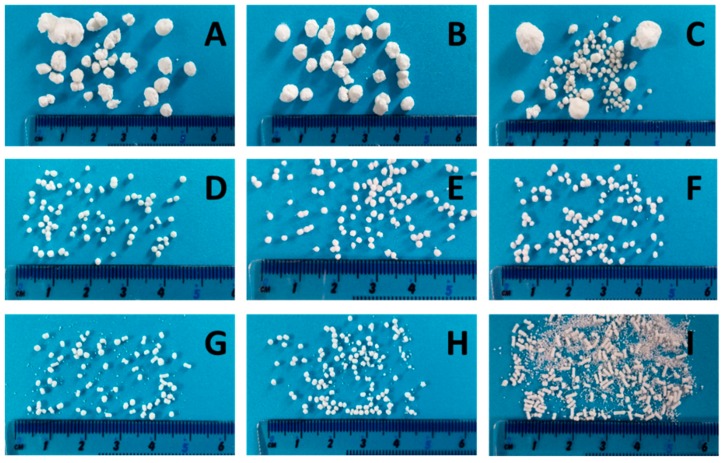
NAM-MCC pellets (80%:20%) after extrusion and spheronisation (3 min, 15 g) with different solid feed rates (g/min): 5.3 (**A**), 5.5 (**B**), 5.6 (**C**), 6.2 (**D**), 6.4 (**E**), 6.6 (**F**), 6.7 (**G**), 7.4 (**H**), 7.9 (**I**). The granulating liquid was distilled water with a constant feed of 1.74 g/min.

**Figure 3:**
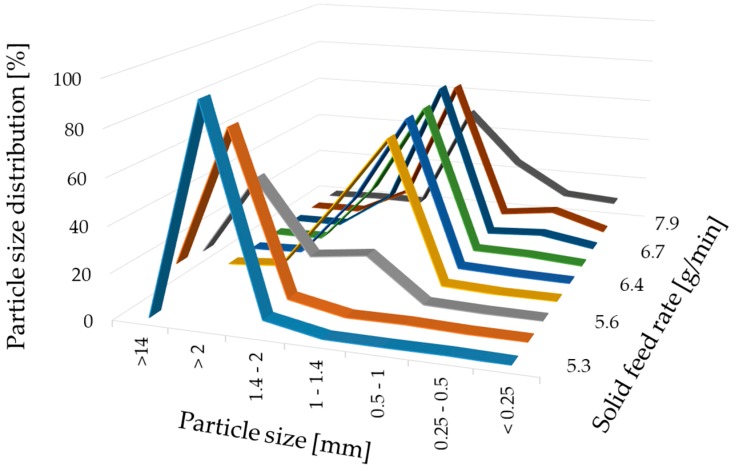
Particle size distribution (%) after sieve analysis of NAM-MCC pellets (80%:20%) after extrusion and spheronisation (3 min, 15 g) with different solid feed rates (g/min). The granulating liquid was distilled water with a constant feed of 1.74 g/min.

For a high vitamin load, a ratio of 80% *w*/*w* NAM and 20% *w*/*w* MCC was chosen. In order to get a paste that can be spheronised without blocking the die of the extruder, the range of the solid-to-liquid ratio had to be evaluated before a systematic investigation. Distilled water was added to the extruder with a constant rate of 1.74 g/min. The solid feed rate was increased from 5.3 g/min to 7.9 g/min to evaluate the particle shape changes (Figure 2). 15 g of the formed extrudates were spheronised for 3 min, dried and photographed. A low solid feed rate resulted in big and irregular agglomerates (A–C), whereby with increasing solid feed rates the amount of fines and dumbbell-shaped particles or strands increased (G–I). The optimal solid feed rate was between 6.2 g/min and 6.6 g/min, which led to spherical particles (D–F). The differences regarding the particle size distribution of different solid feed rates were confirmed by sieve analysis (Figure 3). The lower the solid feed rate, the higher the amount of large particles (>14 mm and >2 mm). The higher the solid feed rate, the higher the amount of fine particles (<1 mm). The highest amount of the useable particles (1–2 mm) was obtained for a solid feed rate of 6.2–6.7 g/min.

**Figure 4:**
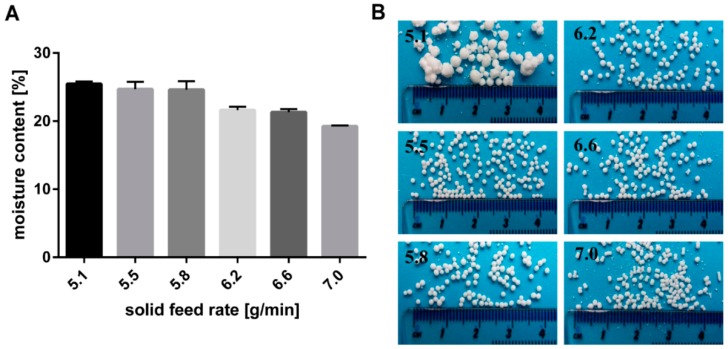
(**A**) Effect of solid feed rate (g/min) on moisture content of wet extrudates (NAM: MCC; 80%:20%) and (**B**) the resulting shape of spheronised particles. The granulating liquid was distilled water with a constant feed of 1.74 g/min.

The effect of the solid feed rate on the moisture content of the extrudates is illustrated in Figure 4. Furthermore, the resulting particles formed after spheronisation are shown in Figure 4. A low solid feed rate resulted in big and irregular particles due to a high moisture content of 25.5%. The wet extrudates tended to stick together during spheronisation and formed irregular and large particles, like a “rolling snowball”. A low moisture content (19.2%) produced brittle extrudates that formed dumbbell-shaped particles. Only in a narrow water content range between 24.7% and 21.3% (corresponding to a solid feed rate of 5.5 g/min and 6.6 g/min respectively), spherical particles were generated. It is known that the moisture content of extrudates influences the spheronisation success. The extrudates must be brittle enough to be broken down into short strands during the spheronisation, but not so friable, that they disintegrate completely. Furthermore, the material has to be sufficiently plastic, that the broken strands can be rolled into spheres by the action of the friction with the plate. However, if the material is too wet, lumps will be formed during spheronisation [24,25,30]. The range of water level over which spherical pellets were formed is influenced by the incorporated drug characteristics. It is documented that the ideal water level decreased with increasing water solubility of the drug, due to loss of solid matter by dissolution of the drug [8,31]. The optimal water content for glucose, which has the same water solubility as NAM (1000 g/L), was 30.0% vs. 23.6% for NAM. The difference is explained by the higher amount of drug used in the present study (80%) compared to others, who used 50% drug and 50% MCC [8]. Decreasing the MCC fraction to a lower concentration as 50% (*w*/*w*) can lead to negative effect on the process yield and on the pellet properties [9]. As shown in the present study, Podczeck et al. also showed pellets with good properties using 20% MCC and 80% drug (lactose, ≈195 g/L water solubility). The optimal water content was 23.66% for a formulation with 80% lactose. A lower water content (21.66%) was required for the more water-soluble ascorbic acid (≈400 g/L) [32].

### 3.2. Design of Experiment—Extrusion Process

**Figure 5:**
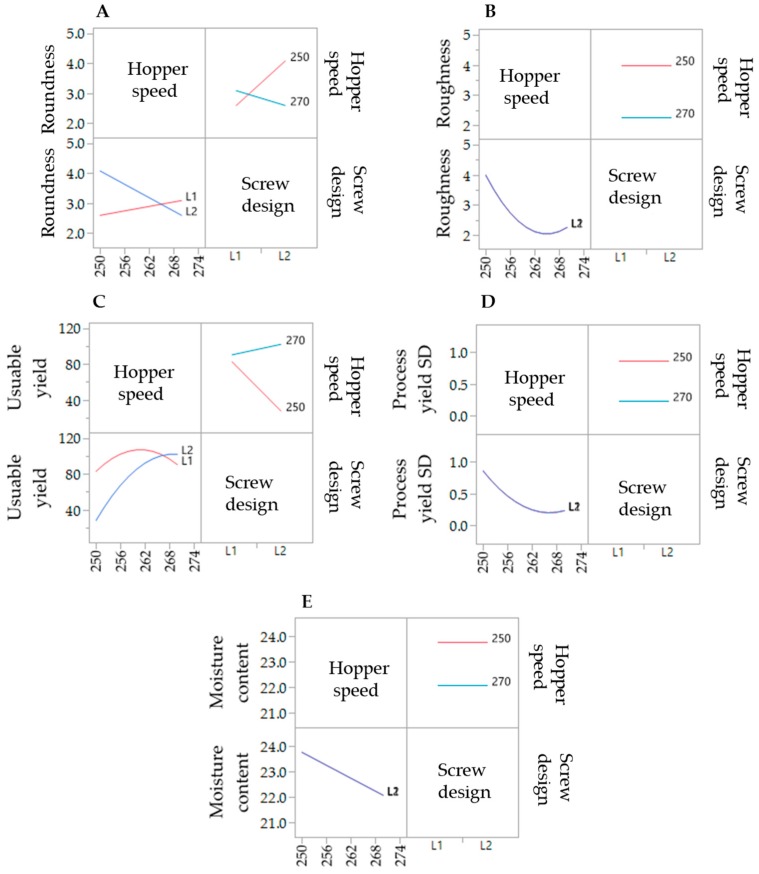
Interaction profiles of the extrusion process variables hopper speed and screw design on the responses roundness (**A**), roughness (**B**), useable yield (**C**), process yield SD (**D**) and moisture content (**E**).

**Figure 6:**
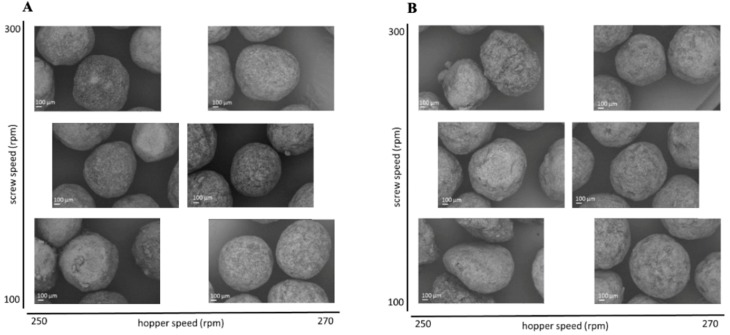
SEM pictures of spheronised NAM: MCC-particles after DoE for extrusion process with screw design L1 (**A**) and L2 (**B**) with varying screw speed (*y*-axis) and hopper speed (*x*-axis).

**Figure 7:**
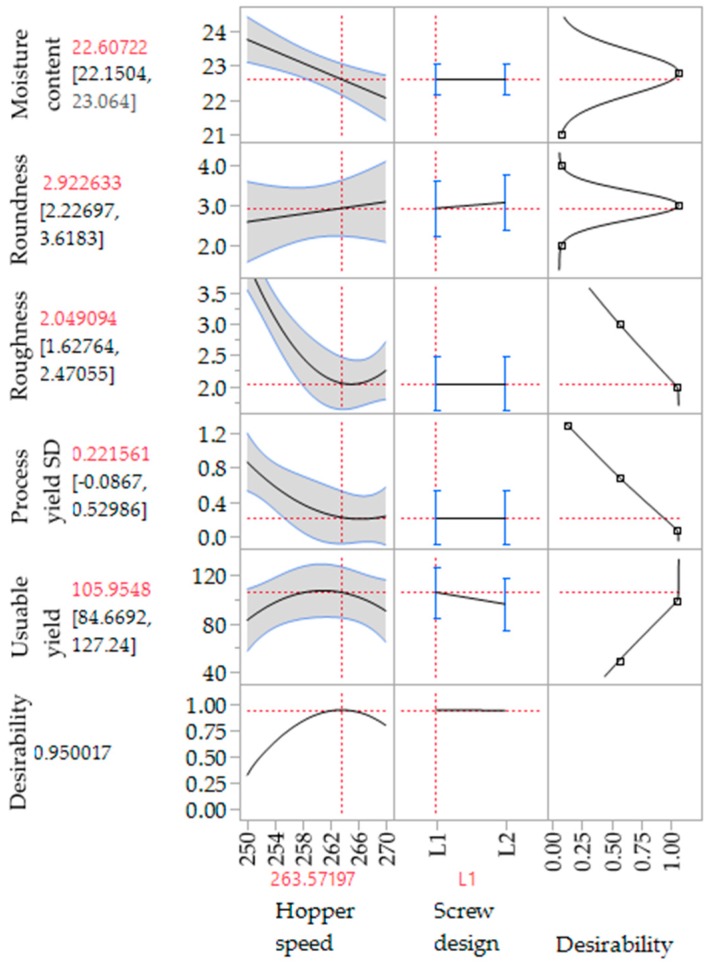
Proposed extrusion configurations for a maximized desirability of all responses (CQAs) measured (useable yield, process yield SD, roughness, roundness, moisture content).

To evaluate the effect of the extrusion process parameters on the particle characteristics, the parameters for the spheronisation process (load: 15 g, rotation speed: 75%, time: 9 min) as well as the formulation composition were held constant. During the extrusion process the factors screw design, screw speed and hopper speed (= solid feed rate; powder feed rate (g/min) = 0.0382 × hopper speed − 3.6991) were varied. The effect of the above factors on the responses such as moisture content and process yield variations (SD of process yield) of the extrudates and roundness, roughness and useable yield of the spheronised particles was examined using a DoE approach. The coefficient of determination (*r*^2^) varied in a broad range depending on the responses. The *r*^2^ of the roughness (0.845) and the useable yield (0.817) indicated a good fitting between the independent variables and responses in the model, in contrast to the moisture content (0.577), the process yield SD (0.544) and the roundness (0.470). However, only the model for the prediction of the roundness gave no significant results (*p* = 0.1471). The outcomes of all tested combinations are shown in Table 1. The changes on the screw speed values showed no significant effect on all responses measured. Therefore, the screw speed was excluded from the model for a better predictability. The hopper speed had a significant effect on the roughness (*p* = 0.0002) and the useable yield (*p* = 0.0087) of the particles as well as on the process yield variations (*p* = 0.0154) and the moisture content (*p* = 0.0041). Furthermore, the combination of hopper speed and screw design influenced the useable yield significantly (*p* = 0.0221). The interaction profiles between screw design and hopper speed on the responses are shown in Figure 5. As can be seen, there was no interaction between screw design and hopper speed for the responses’ roughness of the particles, moisture content and process yield of the extrusion (B, D, E). A lower hopper speed, however, led to an increased roughness, a higher moisture content and a higher variability of the process yield (B, D, E). There was a clear interaction between screw design and hopper speed for the responses’ roundness and useable yield (A, C). The useable yield of the particles was relatively constant and independent from the screw design when using a hopper speed set to 270 (6.6 g/min). The lower hopper speed set to 250 (5.8 g/min) in combination with screw design L2 resulted in a drastic decrease of the yield (C). This result was in accordance with the roundness of the particles: the higher hopper speed (270) led to more spherical particles in combination with screw design L1 (A). SEM pictures of all runs are shown in Figure 6. They were used to assess the roundness and roughness of the particles. In general, it is seen that the screw design L2 always resulted in more irregular shaped particles. Modelling a maximisation of the desirability (0.95) of all responses, a combination of the screw design L1 and a hopper speed set to 263 was proposed (Figure 7).

In the present study, a lower hopper speed (=higher water content) resulted in a higher roughness, although a higher density was documented for a higher water content after spheronisation [33]. However, it is reported that for twin-screw granulation a higher powder feed leads to an increased channel fill and torque, which increases the strength and decreases the porosity of the resulting granules [34]. This could be an explanation for the observed lower roughness of the spheronised particles of the present study using a higher powder feed. Furthermore, the implementation of kneading elements can result in denser granules after extrusion [35], which was not shown in the present results. The screw speed showed no significant effects on the particle characteristics, although the screw speed can also influence the barrel fill and the density of the extrudates. However, if the screw speed was too low, blockages were observed, which is in accordance with a high fill level and an increased material compaction [35,36]. The roundness of the particles was not significantly affected by variations of the extrusion process, which could be related to a higher impact of the spheronisation process and the moisture content [37,38] and will be discussed later. Whereby, the moisture content is significantly influenced by the hopper speed, due to an independent feed of the powder and the granulating liquid.

### 3.3. Design of Experiment—Spheronisation Process

**Figure 8:**
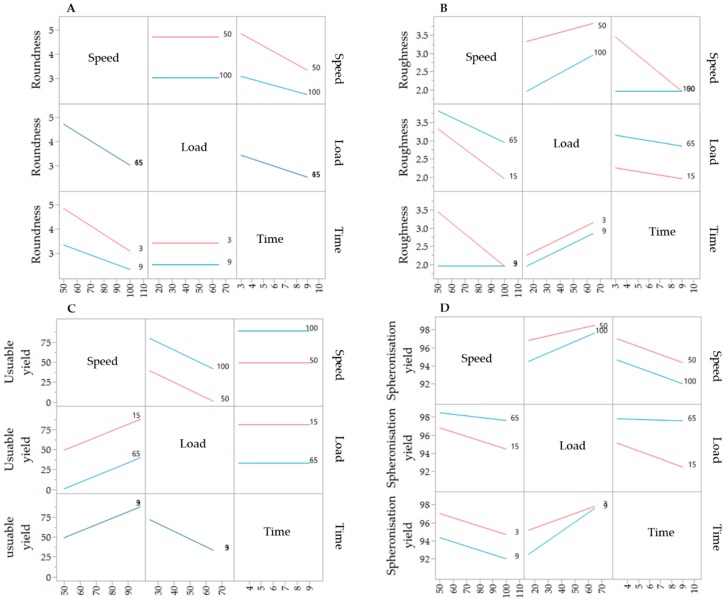
Interaction profiles of the spheronisation process variables rotation time, speed and load on the responses roundness (**A**), roughness (**B**), useable yield (**C**) and spheronisation yield (**D**).

The results of the DoE for the spheronisation process are shown in Table 2. The extrusion process was done at the following constant conditions: screw design L1, screw speed of 130 rpm and a hopper speed set to 270 (6.6 g/min). During spheronisation, the effects of the factors load, speed and time on the roundness, roughness, useable yield and spheronisation yield were investigated. Compared to the extrusion process, the spheronisation process showed a better fitting between the independent factors and the responses, which is demonstrated by higher *r*^2^ of the roughness (0.976), spheronisation yield (0.958), roundness (0.741) and useable yield (0.614). The spheronisation load had a significant effect on the useable yield (*p* = 0.0380), the roughness (*p* = 0.0026) and the spheronisation yield (*p* = 0.0016), whereby the speed affected significantly the roundness (*p* = 0.0202), roughness (*p* = 0.0111) and the spheronisation yield (*p* = 0.0238). The rotation time showed significant influences on the roundness (*p* = 0.0426) and roughness (*p* = 0.0026) of the particles and the spheronisation yield (*p* = 0.0321). Furthermore, the combination of speed and time significantly affected the particle roughness (*p* = 0.0026). The interaction profiles of the factors load, speed and time are exhibited in Figure 8. Regarding roundness, a higher speed always resulted in rounder particles, independently of the load. A low spheronisation time leads to more dumbbell-shaped particles and strands, especially in combination with a low speed. However, this effect was reduced in combination with a high speed. If the rotation time was too long, while rotating with a speed of 100%, the resulting particles were more irregular (A). The roughness of the particles was increased by increasing load and due to a low speed in combination with a low rotation time (B). The useable yield is only affected by the rotation speed and load: the yield was always higher with the highest speed and the lowest load (C). The spheronisation yield was dependent on all process parameters, whereby a high load, a low speed and a low time resulted in the lowest abrasion of the particles and therefore in the highest spheronisation yield (D).

**Figure 9:**
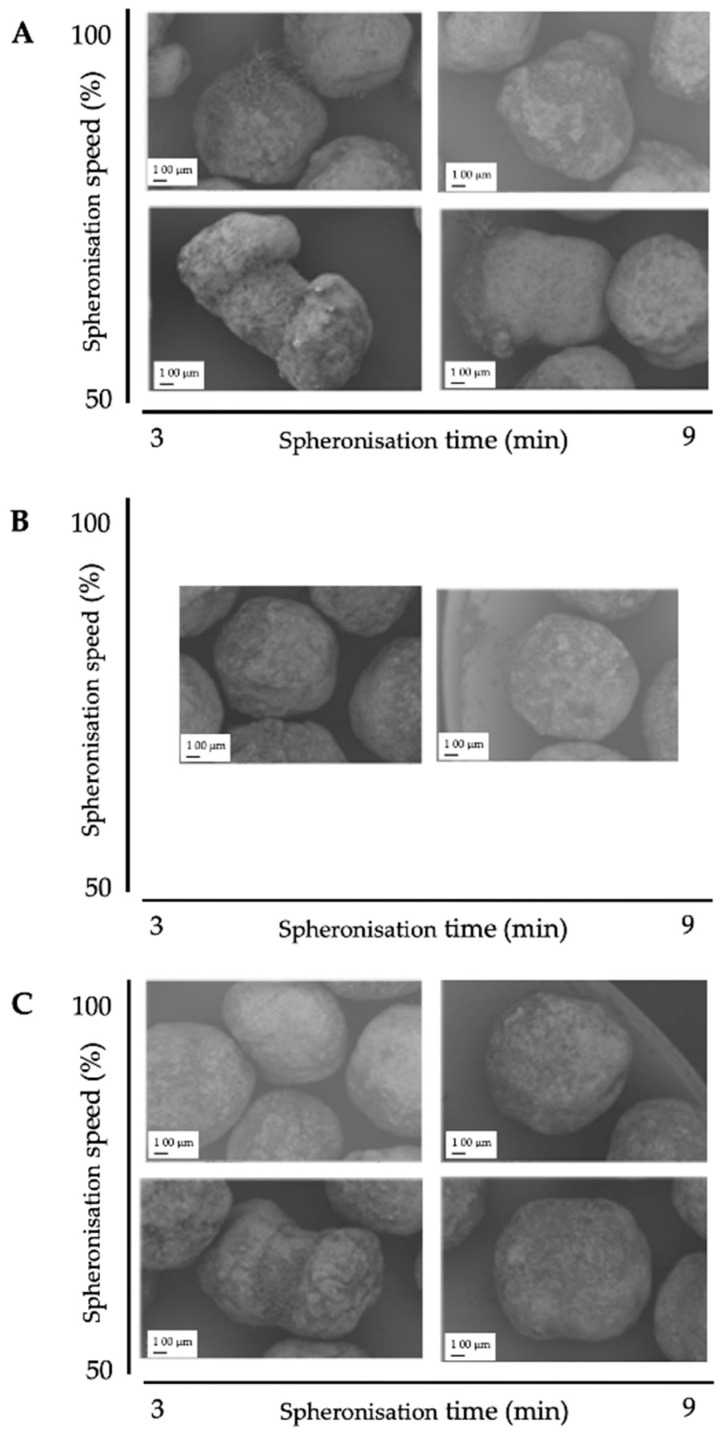
SEM pictures of spheronised NAM: MCC-particles after DoE for spheronisation process with the highest spheroniser load (65 g) (**A**), midpoint of the spheroniser load (40 g) (**B**) and the lowest spheroniser load (15 g) (**C**) with varying spheronisation speed (*y*-axis) and time (*x*-axis).

**Figure 10:**
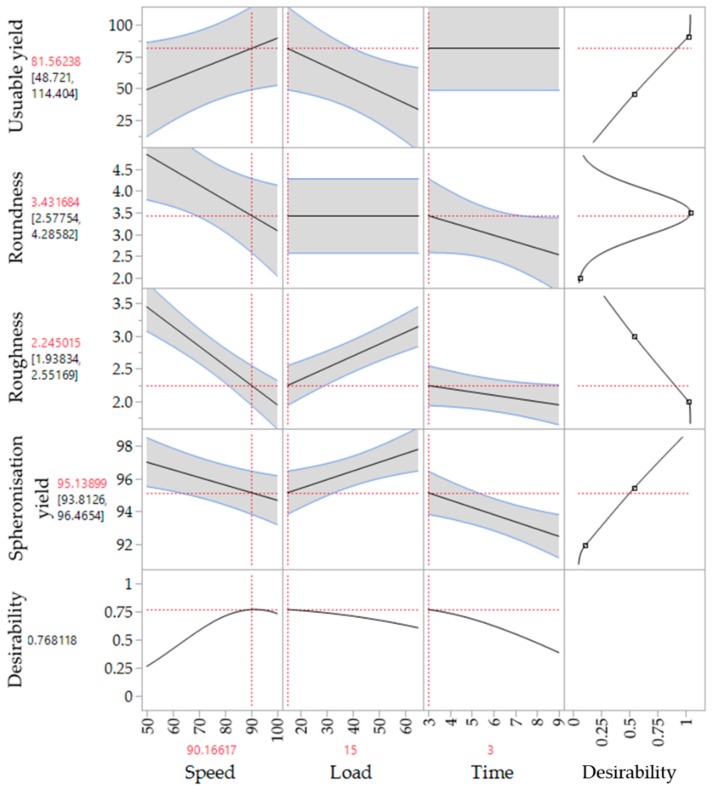
Proposed spheronisation configurations for a maximized desirability of all responses.

The corresponding SEM pictures of the ten runs (Figure 9), show irregular particles due to a high load and dumbbell-shaped particles after spheronisation with a low speed. In comparison to the extrusion process, it is demonstrated that the spheronisation process has a higher impact on the desired responses (CQAs) of the particles. For a maximisation of the desirability (0.77) of all responses, a combination of a load of 15 g, a rotation time of 3 min and a rotation speed of about 91% is proposed after modelling (Figure 10). The effect of the spheronisation conditions on the pellet characteristics has often been evaluated and always showed a great impact, which is in line with the findings of the present study. As expected, the spheronisation yield (remained quantity after abrasion during spheronisation) was affected by all three tested parameters time, speed and load. The lower the speed, the higher the load and the lower the time, the lower would be the particle-plate interactions, which lead to abrasions and loss of material [39,40,41]. The particle-plate and particle-particle interactions are relevant for the roundness, roughness and useable yield of the spheronised particles. The spheronisation process depends on friction between particles-particles and particles-plate. Low speeds resulted in low interactions and therefore in failure of rounding the particles [41] which is in accordance with the present results, where the particles were still strands or dumbbell-shaped. Other authors also documented a high impact of the spheronisation speed on the roundness of the particles, whereby an increase of spheronisation speed resulted in a higher pellet sphericity [22,39,40,42]. A recent article showed that an initial high-speed spheronisation was required to reduce the extrudates. Followed by a medium speed to minimize the fines but to ensure enough round off of the extrudates [43]. Furthermore, for a low water content, the spheroniser speed became more important than the time [39]. Whereby, the time also played a major role for the roundness of the particles also in dependence of the speed [39,44]. In the present study, a longer rotation time led to more spherical particles at a low speed, compared to a shorter time, but this relationship was statistically not significant. The load had no significant effect on the roundness with is in accordance with Krueger et al. [42]. However, for a higher load a longer spheronisation time was needed to form round particles [41], which makes an adjustment of the spheroniser speed, time and load indispensable. Additionally, the roughness of the particles was significantly influenced by spheroniser speed and time. Especially, a high rotation speed for a short time resulted in the lowest roughness of the particles. It is documented, that the spheronisation speed has to be optimised to obtain the desired densification of the particles, which can be insufficient for low speeds [30,41] and would explain the higher roughness at low speeds in the present study. However, contrary results were shown regarding the rotation time. Krueger et al. reported a lower porosity and denser particles with increasing times, whereby the reduction of the porosity was lower, when the pellets consisted of the drug chloramphenicol instead of lactose-monohydrate [42]. Therefore, the higher surface roughness after longer spheronisation times could also be related to the encapsulated NAM.

### 3.4. Verification of the Spheronisation Model

As already mentioned, the coefficient of determination (*r*^2^) varied according to the responses useable yield (*r*^2^ = 0.614), spheronisation yield (*r*^2^ = 0.958), roughness (*r*^2^ = 0.976) and roundness (*r*^2^ = 0.741). The obtained equations for these responses in term of the used factors (rotation speed, time and load) are as follows:

Spheronisation yield=96.226(intercept)−0.7237(time)−0.7962(speed)+1.711(load)+0.6087(time×load)+0.37125(speed×load)

Useable yield=45.187(intercept)+20.2925(speed)−24.065(load)

Roughness=2.7(intercept)−0.375(time)−0.25(speed)+0.375(load)+0.375(time×speed)+0.125(speed×load)

Roundness=3.4(intercept)−0.5625(time)−0.6875(speed)+0.1875(time×speed)

**Figure 11:**
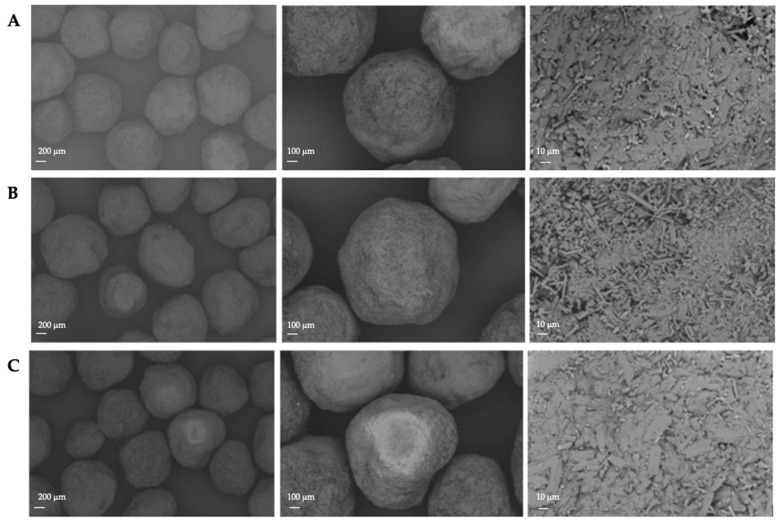
SEM pictures of spheronised particles (NAM: MCC) of three different runs (**A**–**C**) with the same process parameter constellation. Extrusion: screw design L1, screw speed 200 rpm, hopper speed set to 270 (6.6 g/min); Spheronisation: speed 100%, load 15 g, time 3.5 min. Magnification: 50-times, 100-times and 1000-times from left to right.

For the practical verification of the calculated spheronisation model, the following extrusion and spheronisation conditions were used to achieve a high desirability (0.70) of the measured responses: screw design L1, screw speed 200 rpm, hopper speed set 270 (6.6 g/min), rotation speed of 100%, rotation time of 3.5 min and a load of 15 g. Therefore, only the spheronisation model was verified due to similar extrusion conditions as used for the DoE. The run was conducted for about 3 h. Every 15 min, the moisture content of the extrudates was measured. The mean was 23.6% ± 0.58% for 13 measurements in total. The process yield of 44 measurements during the process was 7.95 g/min ± 0.65 g/min, which confirmed a constant process. Furthermore, all runs resulted in well-formed particles with a spherical shape (roundness: 2.5) and a low roughness of 2 (Figure 11). In comparison, the model proposed a roundness of 3 and a roughness of 1.95. The mean useable yield of three different runs was 91.61% ± 1.83% compared to the predicted 89.54%. Thus, the model prediction of the spheronisation process is in good accordance with the measured values.

### 3.5. Comparison of NAM Pellets Produced by Wet Extrusion-Spheronisation and Spray Granulation

**Figure 12:**
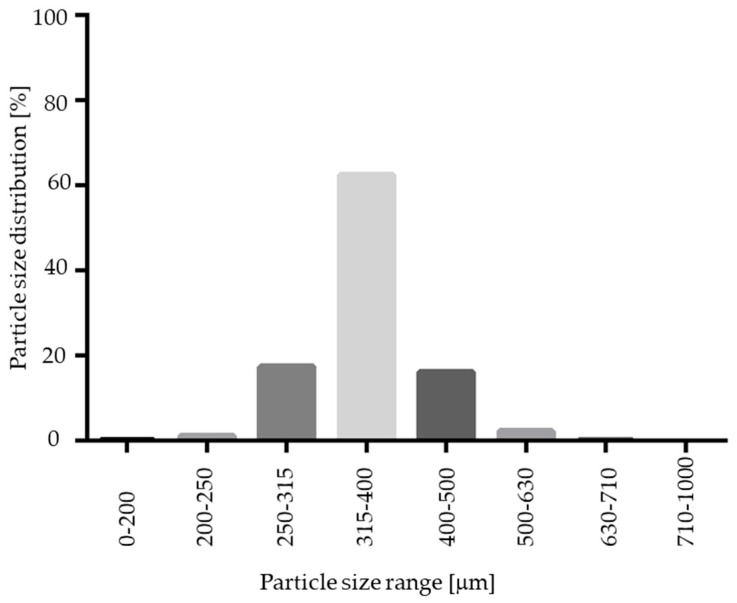
Particle size distribution of spray granulated NAM pellets.

**Figure 13:**
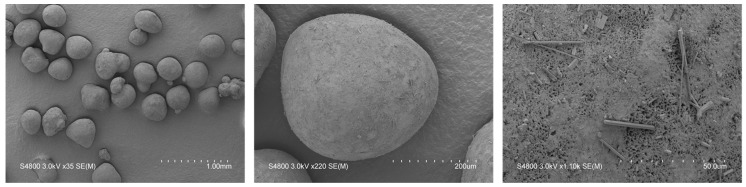
SEM pictures of NAM pellets produced by spray granulation.

As indicated in Figure 3 and Figure 12, the resulting pellet diameter is bigger for NAM extrudates than for NAM spray granulates. This is due to the die diameter of 1 mm for the extruder. A smaller die diameter would reduce the particle diameter. However, the useable size of the NAM pellets produced by spray granulation was in a narrow range between 315 µm and 400 µm [15]; bigger particles were irregular and therefore not applicable for the coating process. The useable yield of the spray granulated particles was about 63% (Figure 12) and lower compared to the spheronised NAM extrudates (92%). Using a high drug load of 96% in the spray granulated pellets, the conditions were optimized in pre-experiments (data not shown) to achieve a high yield, spherical particles and a continuous and stable granulation process. Possibly, the coating of a NAM-HPMC-mixture on spherical cores (like Cellets^®^) could lead to a higher yield but would also reduce the relative load of NAM. Furthermore, as shown by SEM pictures in Figure 13, the spray granulated NAM pellets showed some irregularities in their shapes. The crystalline structure of NAM is clearly demonstrated, whereby the roughness is less pronounced compared to the extrudates, although a higher concentration of NAM is present in the granules (94% vs. 80%). This suggests a higher compactness of the pellets, which were produced by spray granulation.

**Figure 14:**
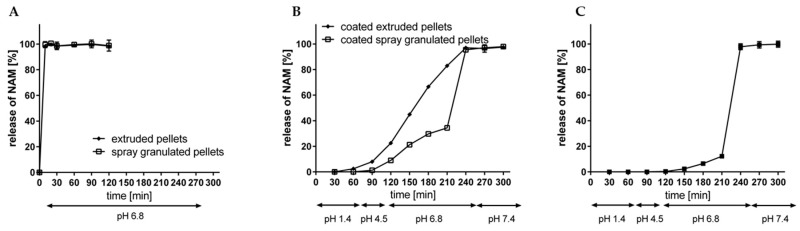
Release rate of NAM from (**A**) uncoated spray granulated and extruded NAM and (**B**) from triple shellac coated spray granulated and extruded NAM pellets with a coating thickness of 3.7/4.9/6.2 mg/cm^2^ and 3.6/4.6/6.0 mg/cm^2^ and (**C**) from triple shellac coated extruded NAM pellets with a coating thickness of 5.8/7.6/10.0 mg/cm^2^ at simulated gastrointestinal conditions (mean ± SD; *n* = 2).

After the extrusion-spheronisation process, the resulted pellets were evaluated regarding their dissolution profile without and with an enteric coating, according to Fangmann et al. [15] (Figure 14A,B). In a simulating fluid with a pH value of 6.8, both, the spray granulated as well as the extruded uncoated NAM pellets liberated NAM immediately within 10 min. Thus, no delay of NAM is attributed to the usage of MCC or HPMC in the formulation as it is described for poorly water-soluble compounds [24,45]. This is mainly explained due to the very high water solubility of NAM (1000 g/L). After the application of the shellac coating with an intermediate citric acid sub-coating on the NAM extrudates, the release of NAM was clearly delayed in simulated gastrointestinal fluids (pH 1.4–7.4) (Figure 14B). The modified shellac coating prevented a premature release at gastric conditions (2.5% after 60 min at pH 1.4), followed by a continuous release at pH value 6.8 (from 22.5% after 30 min to 82.9% after 120 min) and a complete release is reached at a pH value of 7.4. The release profile of the spray granulated NAM pellets with a comparable coating level showed a slower release profile at pH 6.8 (up to 34.4% after 210 min in total) (Figure 14B) [15]. This could be related to the higher roughness of the extruded pellets, which increases the porosity of the coating layer and therefore increases the release rate of NAM. A 1.6-fold higher coating thickness (5.8 mg/cm^2^; 7.6 mg/cm^2^ and 10.0 mg/cm^2^) of the extruded NAM pellets (Figure 14C), resulted in a slower release at pH 6.8 (up to 12.2% after 210 min in total). The exact accordance of the release profile of coated extruded and spray granulated particles would be existing with a coating layer in between. However, both processes are suitable for the production of NAM pellets and a further uniform coating. Furthermore, the spray granulation process as well as the twin-screw extrusion can be easily scaled up and performed as continuous processes. On the one hand, the narrow water level range (<10%) may cause problems for further scale-up processes for wet extrusion and spheronisation [8]. On the other hand, the release profile indicates that lower shellac amount is required for a targeted release. Only 3.6% (*w*/*w*) coating weight gain was needed for the application of a coating level of 1 mg/cm^2^, compared to 12% (*w*/*w*) for the spray granulates. Related to 100 g NAM, the required addition of shellac was reduced from 60 g to min. 27.5 g for spray granulated compared to wet extruded NAM pellets, respectively. Due to the resulting thicker coating level for extruded pellets, it is proposed that the required amount could be further decreased (max. to 16.5 g of shellac per 100 g NAM).

## 4. Conclusions

All in all, the extrusion-spheronisation process demonstrates an opportunity to produce highly loaded NAM pellets with a useable yield of about 90%. The pellets were spherical and had a rough surface. In comparison to spray granulated NAM pellets, the extruded NAM pellets resulted in a higher roughness and the useable yield was increased. Finally, the coating and dissolution test showed that the extruded and spheronised pellets are also suitable for a protective coating with an ileocolonic release profile in vitro. The quality by design (QbD) proved to be a suitable method to better understand the effects of the critical process parameters (CPPs) on the critical quality attributes (CQAs) of the desired product in an efficient way. It was shown, that the prediction model was in good agreement with the measured outputs. The design of experiments (DoE), showed the spheronisation conditions speed, time and load had a greater impact on the CQAs of the pellets than the extrusion conditions screw design, screw speed and solid feed rate (hopper speed). The roundness was significantly affected by the rotation speed and time, whereby the roughness varied depending on the spheroniser load, time, speed time and the solid feed rate. Furthermore, the spheroniser load, solid feed rate and the solid feed rate screw design significantly impacted the useable yield. The screw speed showed no significant effect. The moisture content is one of the most critical factors for an optimal spheronisation result. Therefore, the moisture content need to be adjusted to the formulation characteristics to ensure an optimal product. The spheronisation conditions must be adjusted using a multivariate approach.

## Figures and Tables

**Table 1:** Design of Experiment of the extrusion process and the resulted responses (for all runs the spheronisation process was constant with 9 min time, 75% speed and 15 g load).

Run	Pattern	Screw Design	Screw Speed [rpm]	Hopper Speed ^1^ [rpm]	mc [%]	Roundness	Roughness	py SD	uy [%]
**1**	100	L1	200	260	23.87	3	2	0.08	91.62
**2**	1+−	L1	300	250	23.86	3	4	1.19	91.18
**3**	1−−	L1	100	250	23.79	2	4	0.41	86.62
**4**	1++	L1	300	270	21.70	3	2	0.23	95.79
**5**	100	L1	200	260	23.55	3	3	0.12	98.12
**6**	1−+	L1	100	270	21.39	3	2	0.23	97.53
**7**	200	L2	200	260	23.15	2	2	0.36	98.04
**8**	2−−	L2	100	250	24.09	5	4	1.3	0
**9**	2+−	L2	300	250	22.78	4	4	0.56	42.42
**10**	2++	L2	300	270	21.85	3	3	0.26	95.96
**11**	200	L2	200	260	22.12	3	2	0.65	97.49
**12**	2−+	L2	100	270	22.78	3	2	0.22	96.43

^1^ Hopper speed set to 250, 260 and 270 resulted in a feed rate of 5.8 g/min, 6.2 g/min and 6.6 g/min. mc = moisture content; py = process yield; uy = useable yield.

**Table 2:** Design of Experiment of the spheronisation process and the resulted responses (for all runs the extrusion process was constant with screw design L1, 130 rpm screw speed and 270 rpm hopper speed).

Run	Pattern	Time [min]	Speed ^1^ [%]	Load [g]	uy [%]	Roundness	Roughness	sy [%]
**1**	+−+	9	50	65	0	4	2.5	97.62
**2**	−++	3	100	65	27.05	3.5	3	97.00
**3**	+−−	9	50	15	71.43	3	2	94.27
**4**	−−−	3	50	15	0	5	3.5	96.93
**5**	++−	9	100	15	90.99	3	2	91.93
**6**	000	6	75	40	65.29	2.5	2.5	96.28
**7**	−−+	3	50	65	0	5	4	98.94
**8**	−+−	3	100	15	86.44	3	2	94.60
**9**	000	6	75	40	81.38	3	2.5	96.83
**10**	+++	9	100	65	29.29	2	3	97.86

^1^ rotation speed of 50%, 75% and 100% are in accordance with 430 m/min, 680 m/min and 900 m/min. uy = useable yield; sy = spheronisation yield.
